# Content reporting of exercise interventions in rotator cuff disease trials: results from application of the Consensus on Exercise Reporting Template (CERT)

**DOI:** 10.1136/bmjsem-2019-000656

**Published:** 2019-12-22

**Authors:** Daniel H Major, Yngve Røe, Margreth Grotle, Rebecca L Jessup, Caitlin Farmer, Milada Cvancarova Småstuen, Rachelle Buchbinder

**Affiliations:** 1 Faculty of Health Sciences, Oslo Metropolitan University, Oslo, Norway; 2 Research and Communication Unit for Musculoskeletal Health, Oslo University Hospital, Oslo, Norway; 3 Cabrini Institute, Monash Department of Clinical Epidemiology, Melbourne, Victoria, Australia; 4 Monash University Department of Epidemiology and Preventive Medicine, Melbourne, Victoria, Australia

**Keywords:** exercise, shoulder, reporting guidelines, publication quality

## Abstract

**Background:**

Exercise interventions are frequently recommended for patients with rotator cuff disease, but poor content reporting in clinical trials of exercise limits interpretation and replication of trials and clinicians’ ability to deliver effective exercise protocols. The Consensus on Exercise Reporting Template (CERT) was developed to address this problem.

**Objective:**

To assess completeness of content reporting of exercise interventions in randomised controlled trials for patients with rotator cuff disease and the inter-rater reliability of the CERT.

**Design:**

Critical appraisal.

**Methods:**

Independent pairs of reviewers applied the CERT to all 34 exercise trials from the most recent Cochrane Review evaluating the effect of manual therapy and exercise for patients with rotator cuff disease. We used the CERT Explanation and Elaboration Statement to guide assessment of whether each of the 19-item criteria were clearly described (score 0–19; higher scores indicate better reporting). Percentage agreement and the prevalence and bias adjusted kappa (PABAK) coefficient were used to measure inter-rater reliability.

**Results:**

The median CERT score was 5 (range 0–16). Percentage agreement was high for 15 items and acceptable for 4 items. The PABAK coefficient indicated excellent (5 items), substantial (11 items) and moderate (3 items) inter-rater agreement.

**Conclusion:**

The description of exercise interventions for patients with rotator cuff disease in published trials is poorly reported. Overall, the inter-rater reliability of the CERT is high/acceptable. We strongly encourage journals to mandate use of the CERT for papers reporting trial protocols and results investigating exercise interventions.

**Recommendations for future research**

- Evaluate the effect of journal implementation of the Consensus on Exercise Reporting Template (CERT) reporting guideline on the quality of the content reporting of exercise interventions.
- Determine which CERT items are/are not essential to intervention success in trials investigating the value of exercise.
- Further refinement and evaluation of use of the CERT to better develop and describe exercise interventions and their important components.

**What is already known?**

- Exercise interventions are recommended as a first-line treatment for people with rotator cuff disease, although there is low-quality evidence that exercise interventions may provide limited benefits.
- The content reporting of complex interventions, such as exercise interventions, are often poorly reported, which limits interpretation and replication of trials and clinicians’ ability to deliver effective exercise protocols where they exist.
- Early studies have demonstrated that the CERT, developed to address the problem of incomplete reporting of exercise interventions, may be of value for evaluating exercise interventions in musculoskeletal trials.

**What are the new findings?**

- The completeness of content reporting of exercise intervention in randomised controlled trials of trials for people with rotator cuff disease is poor.
- The CERT is a reliable tool to evaluate the completeness of reporting of exercise interventions in trials.

## Introduction

Shoulder pain is a prevalent and often long-lasting complaint.^1 2^ Presentation for shoulder pain has an incidence of 10 per 1000 in primary care^3 4^ and prevalence of 7%–26%.^2^ People with shoulder disorders report experiencing pain, physical function/activity limitations, participation restriction, sleep disruption, cognitive dysfunction, emotional distress and other pathophysiological manifestations (other than pain).^5^ The most common subgroup of shoulder pain is rotator cuff disease,^3 6^ an umbrella term for various disorders of the rotator cuff, such as subacromial impingement syndrome, rotator cuff tendonitis or tears, and subacromial bursitis. Exercise interventions have been recommended as a first-line treatment for patients with rotator cuff disease.^7^ However, systematic reviews have reported conflicting conclusions about their effectiveness in reducing pain and disability.^8–11^

We know from previous studies that where description of interventions are available, they seldom provide the level of detail required for other researchers to validate trials through replication.^12–18^ Inadequate content reporting of interventions also limits the clinician’s ability to interpret the study findings and to deliver an effective exercise protocol in their clinical practice,^19^ and means it may not be possible to determine which specific components of exercise interventions may be associated with better (or worse) outcomes.^20 21^ So far, no studies have been conducted to specifically assess the content reporting of exercise interventions investigated in clinical trials for patients with rotator cuff disease.

In order to improve the content reporting of interventions, the Template for Intervention Description and Replication (TIDieR)^22^ checklist was developed. However, this guide was not specifically designed for exercise interventions and does not cover all important exercise prescription domains. The Consensus on Exercise Reporting Template (CERT) was developed to specifically address the problem of incomplete reporting of exercise interventions.^23 24^ Based on evidence from a systematic review^12^ and subsequent international Delphi consensus study that included 137 experts, it can be used to both review existing published trials of exercise interventions and act as a template when designing and evaluating exercise interventions.^24^

The main objective of this study was to assess the content reporting of exercise interventions in randomised controlled trials (RCTs) for patients with rotator cuff disease by applying the CERT. The secondary objective was to assess the inter-rater reliability of the 19 CERT items.

## Methods

### Eligibility criteria

We used the recently published 2016 Cochrane Review evaluating the effect of manual therapy and exercise for reducing pain and improving function for patients with rotator cuff disease to identify RCTs for inclusion in this study.^11^ Page *et al*^11^ included RCTs that compared exercise to placebo, no treatment, usual care or another active intervention among adults (≥18 years) with rotator cuff disease. The term ‘rotator cuff disease’ was used in the review for disorders of the rotator cuff labelled and/or defined by the trial authors using terminology such as subacromial impingement syndrome, rotator cuff tendonitis or tendinopathy, supraspinatus, infraspinatus or subscapularis tendonitis, subacromial bursitis or rotator cuff tears. Trials could include interventions provided to participants in any setting (eg, outpatient, at home or in the community) and must have involved the prescription of a supervised or unsupervised exercise programme. The intervention could have been with or without the addition of other components (eg, manipulation, lifestyle modification or counselling).

We included 34 exercise trials reported up to March 2015 from Page *et al*’s Cochrane Review.^11^

### Data extraction guidelines

We used previously described data extraction guidelines to standardise the information that was extracted from each included paper.^23^ Descriptive data were systematically extracted into a spreadsheet, checked for consistency and merged into one document. In order to ensure a similar understanding of the application of the CERT across five reviewers, all reviewers independently pilot tested the data extraction form using one study, which was not included in the final 34 reviewed. All reviewers discussed their CERT ratings on a video conference in pairs with DHM. We estimated the time of the familiarisation process to be approximately 1.5 hours.

### Application of the CERT

Two reviewers independently scored each included study by applying the CERT.^23^ Five reviewers were involved in the application of the CERT (CF, RLJ, YR, MG and DHM). Three reviewers (CF, RLJ and MG) applied the CERT in five trials each; one reviewer (YR) applied the CERT in 19 trials; and another reviewer (DHM) applied the CERT in all included trials. The CERT includes 16 categories and 19 separate items considered essential in the reporting of reproducible exercise interventions listed under seven domains: what (materials), who (provider), how (delivery), where (location), when and how much (dosage), tailoring (what and how) and how well (compliance/planned and actual).^23^ The CERT domains include information about any equipment used for exercises, the exercise instructor, core procedural and contextual elements of the exercise intervention that are required for replication, information about participant motivation strategies and whether, and how well, participants complied with the exercise programme.

A more detailed description of the CERT items is available in the Explanation and Elaboration Statement.^23^ This statement was used to guide the scope and interpretation of each CERT item. Each CERT item was rated as ‘yes’ (criterion met, indicating item clearly reported), ‘no’ (indicating item not reported or not clearly described) or ‘unsure’, and an overall rating of the exercise description was also made. For no or unsure responses, detailed comments about what was missing or what was unclear were recorded. We summed the number of items rated as yes to compute a total score ranging from 0 to 19 (0=no items clearly described to 19=all CERT items clearly described).

If the authors specifically referred to published protocols, online appendices and supplementary data, the reviewers retrieved and extracted these additional data when relevant. The reviewers also recorded whether the study was published in an open access journal and how easy the intervention description was to access (ie, available in the published paper or required additional data from other sources and, if so, whether this was open access).

Following completion of the review by both reviewers, any disagreements were discussed. If agreement could not be reached, an independent arbiter from the research team was to be consulted.

### Risk of bias assessment

Risk of bias assessments of the included trials, based on the Cochrane Risk of Bias Tool,^25^ were taken from the original Cochrane Review.^11^ The following domains were assessed: random sequence generation, allocation concealment, blinding of participants and personnel, and blinding of outcome assessment (subjective and objective). The risk of bias figure was prepared using RevMan V.5.3 (The Nordic Cochrane Centren, Copenhagen)

### Inter-rater reliability

Inter-rater reliability of the CERT was assessed for each of the 19 CERT items (including subitems a and b for items 7, 14 and 16) using percentage agreement^26^ and the prevalence and bias adjusted kappa (PABAK) coefficient.^27^ While kappa statistics measures chance-adjusted agreement and is therefore more robust than simple percentage agreement, when the prevalence of one of the categories is much higher than that of the other, chance agreement will be high and kappa can have unexpectedly low values.^26–28^ For percentage agreement, a score of 70% or greater is considered acceptable and ≥80% is considered high.^28^ For PABAK coefficients, the strength of agreement is interpreted as follows: 0=poor, 0.01–0.20=slight, 0.21–0.40=fair, 0.41–0.60=moderate, 0.61–0.80=substantial and 0.81–1=excellent.^28^

### Data analysis

Data were entered into SPSS V.22 and were analysed using descriptive statistics and narrative summaries. For each study, the total CERT score was presented together with the percentage of a maximum CERT score of 19. The bootstrapped median was calculated using STATA (Version 12. College Station, TX, United States of America). Bootstrapping is a statistical method based on simulation of random sampling from the available data. We have performed 10 000 repetitions of the sampling creating samples with the same statistical properties as the original data set. The estimate of the median and 95% CI were calculated directly from the simulated repeated sampling. In this way, we did not have to assume any statistical distribution for the median and achieved a higher level of precision when constructing the CI.

## Results

**Figure 1:**
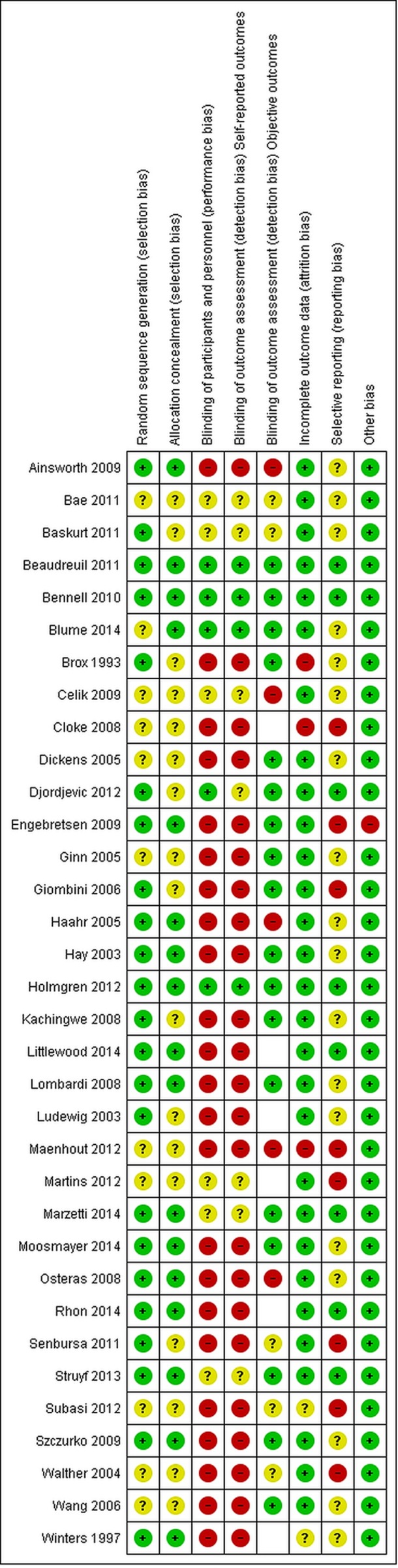
Risk of bias summary: Cochrane Review of authors' judgements about each risk of bias item for each included study. Empty cells mean that objective outcomes were not measured in the trial, so an assessment of the risk of bias due to lack of blinding of such outcomes was not applicable.

Twenty of the 34 trials were open access articles (table 1). The trials were from 15 different countries, and the main components of the exercise interventions most often included strengthening, scapula stabilising, stretching and ‘corrective’ exercises. Twelve trials referred specifically to supplementary material, and five of these were not open access.^29–33^ Of the included trials, three^34–36^ were judged to be at low risk of bias; eight trials^37–44^ were at unclear risk of bias; and 23 trials^45–67^ were at high risk of bias (figure 1).

**Table 1:** Description of the included studies

First author, year	Country	Main components of the exercise intervention	Open access	Needed supplementary material
Ainsworth, 2009	England	Stretching exercises, strengthening exercises, posture correction and adaptation of functional activities	Yes^45^	No
Bae, 2011	South Korea	Motor control and strengthening exercises	Yes^37^	No
Baskurt, 2011	Turkey	Standardised flexibility, strengthening and Codman exercises; group II additionally performed scapular stabilisation exercise	No^38^	No
Beaudreuil, 2011	France	Dynamic humeral centring aimed at lowering the humeral head	No^35^	Online appendix (open access)
Bennell, 2010	Australia	Improving dynamic scapular control, strengthening scapular stabiliser and rotator cuff muscles, improving shoulder and thoracic posture and increasing range of motion of thoracic extension	Yes^34^	Protocol paper^71^ and online appendix (open access)
Blume, 2015	USA	Eccentric and concentric exercises aiming at optimising rotator cuff and scapular muscle recruitment	Yes^42^	Appendix (open access)
Brox, 1993	Norway	Resistance training of the shoulder rotators and scapular stabilising muscles	Yes^39^	No
Celik, 2009	Turkey	Exercises below or above 90°, T-bar (wand) exercises, posterior capsule stretching and internal rotation exercises and rotator cuff strengthening exercises were performed.	Yes^57^	No
Cloke, 2008	England	Kinetic chain exercises, scapular stabilisation, range of motion exercises, closed chain exercises, plyometric exercises and rotator cuff exercises	No^58^	Kibler 1998^31^ (not open access)
Dickens, 2005	England	Exercises for the recruitment and strength of scapulothoracic muscles and rotator cuff	No^46^	No
Djordjevic, 2012	Serbia	Pendulum exercises and pain-limited, active ROM exercises of shoulder elevation, depression, flexion, abduction, rotations and strengthening exercises. Strengthening exercises were isometric in nature, working on the external shoulder rotators, internal rotators, biceps, deltoid and scapular stabilisers.	No^43^	No
Engebretsen, 2009	Norway	Exercises with a principal focus of relearning of normal movement patterns, which could then be transferred to daily activities	Yes^47^	Bøhmer, 1998^29^ (not open access)
Ginn, 2005	Australia	Stretches aimed at lengthening shortened shoulder muscles, exercises aimed at strengthening weakened shoulder muscles, including improving coordination between muscles, and motor retraining aimed at restoring scapulohumeral rhythm during the performance of upper limb tasks	Yes^48^	No
Giombini, 2006	Italy	Pendular swinging in prone position in flexion and extension of the shoulder and passive glenohumeral joint stretching exercises	No^59^	No
Haahr, 2005	Denmark	Exercises aiming at strengthening the stabilisers and decompressors of the shoulder	Yes^49^	No
Hay, 2003	England	Active training of the periscapular muscles and strengthening of the stabilising muscles of the shoulder joint	Yes^50^	No
Holmgren, 2012	Sweden	Strengthening eccentric exercises for the rotator cuff and strengthening concentric/eccentric exercises for the scapula stabilisers	Yes^36^	Online appendix (open access)
Kachingwe, 2008	USA	The exercise programme included posterior capsule stretching, postural correction exercises, and an exercise programme focusing on rotator cuff strengthening and scapular stabilisation	Yes^51^	No
Littlewood, 2014	England	Self-managed loaded exercise using a single exercise	Yes^52^	Development paper^72^ (open access)
Lombardi, 2008	Brazil	Strengthening exercises for the shoulder (flexors, extensors, medial and lateral rotators)	Yes^53^	No
Ludewig, 2003	USA	Stretching of the pectoralis minor and for the posterior shoulder, muscle relaxation of the trapezius and strengthening exercises of external rotators and serratus anterior	Yes^60^	No
Maenhout, 2013	Belgium	Internal and external rotation resisted with an elastic band. The intervention group additionally performed the eccentric phase of full can (thumb up) abduction in the scapular plane with a dumbbell weight	No^61^	No
Martins, 2012	Brazil	Strengthening and stretching exercises were carried out for muscles of the shoulder complex with or without the addition of proprioceptive exercises	Yes^62^	Kuhn, 2009^32^ (not open access)
Marzetti, 2014	Italy	Neurocognitive therapeutic exercises with the aim to teach the patient pathological elements, avoiding compensation and how to rebuild and recover movements in a smooth and functional way. Traditional therapeutic exercise contained strengthening exercises of the rotator cuff and scapular stabilising muscles, stretching exercises, Codman’s pendulum exercises and exercises with elastic band	Yes^40^	No
Moosmayer, 2014	Norway	Individualised exercise treatment aimed at correction of scapula mal positioning at rest and the restoration of ideal scapula positioning and centring of the humeral head during movement	Yes^54^	A Norwegian book* and an appendix
Østerås, 2008	Norway	A combination of aerobic exercises and low-dosage or high-dosage medical exercise therapy	No^63^	No
Rhon, 2014	USA	Exercises directed to the shoulder girdle or thoracic or cervical spine	No^55^	Protocol paper^73^ and appendix (open access)
Şenbursa, 2011	Turkey	Range of motion, stretching and strengthening exercises for the rhomboid, levator scapulae, serratus anterior and rotator cuff muscles	No^56^	No
Struyf, 2013	Belgium	Stretching and motor control training of the scapula Exercise therapy comprised of an eccentric muscle strength training programme of the rotator cuff muscles	No^41^	Mottram, 1997^30^ (not open access)
Subasi, 2012	Turkey	Stretching and range of motion exercises followed by land-based or water-based strengthening exercises	No^64^	No
Szczurko, 2009	Canada	Isometric shoulder strength training and a series of passive, active-assisted and active range of motion, muscle strengthening and joint therapy	Yes^65^	Hagberg, 2000^74^ (open access) Levoska, 1993^33^ (not open access)
Walther, 2004	Germany	Exercises aiming at strengthening the depressor muscles and centring the humeral head	No^66^	No
Wang, 2006	USA	Customised exercises designed by the authors to correct different shoulder classification systems	No^67^	No
Winters, 1997	Netherlands	Exercise therapy	Yes^44^	No

*Not used when assessing the content reporting.

### Final consensus CERT results

The CERT evaluation of the reporting of the exercise interventions of the 34 included trials is shown in table 2. The median score was 5 (range 0–16) out of a possible score of 19. Six trials had a CERT score of ≥10, while the remaining 83% (18/34) all scored <10, indicating that the majority of the details of the exercise interventions was missing from the trial reports. The bootstrapped median with 10 000 bootstrap samples indicated a median CERT score of 5 (95% CI 3.5 to 6.1).

**Table 2:** Results of application of the Consensus on Exercise Reporting Template to each included trial and total number (%) of items fulfilling criteria of acceptable reporting by final consensus

First author, year	1. Exercise equipment	2. Instructor qualifications	3. Individual or group	4. Supervised or not supervised	5. Adherence	6. Motivation	7.a. Progression rule	7.b. Progression description	8. Exercise description	9. Home programme	10. Non-exercise components	11. Adverse events	12. Setting	13.intervention details	14.a. Generic or individually tailored	14.b. Tailored (how)	15. Starting level rule	16.a. Adherence (planned)	16.b. Adherence (actual)	Total number (%) of items fulfilling criteria
Ainsworth, 2009^45^	1	0	0	0	0	0	1	1	0	0	1	0	1	0	1	0	0	0	0	6 (32)
Başkurt, 2011^38^	0	0	0	0	0	0	0	1	0	0	0	0	0	1	0	0	0	0	0	2 (11)
Baskurt, 2011^38^	0	0	0	1	0	0	1	1	0	0	0	0	0	0	0	0	0	0	0	3 (16)
Beaudreuil, 2011^35^	0	0	1	0	1	0	0	1	1	1	1	0	1	1	1	0	0	0	1	10 (53)
Bennell, 2010^34^	1	1	1	1	1	1	0	1	1	1	1	1	1	1	1	1	0	1	0	16 (84)
Blume, 2015^42^	1	0	0	1	0	0	1	1	1	1	0	0	0	0	1	1	1	0	0	9 (47)
Brox, 1993^39^	0	0	0	1	0	0	0	0	0	1	1	0	1	0	0	0	0	0	0	4 (21)
Celik, 2009^57^	0	0	0	1	0	0	0	0	0	1	1	0	0	0	0	0	0	0	0	3 (16)
Cloke, 2008^58^	0	0	0	0	0	0	0	0	0	0	0	0	0	0	0	0	0	0	0	0 (0)
Dickens, 2005^46^	0	0	1	1	0	0	0	0	0	0	1	0	1	0	1	0	0	0	0	5 (26)
Djordjevic, 2012^43^	0	1	0	1	0	0	0	0	0	0	1	0	0	0	0	0	0	0	0	3 (16)
Engebretsen, 2009^47^	0	0	0	1	0	0	0	0	1	0	1	1	1	1	0	0	0	0	0	6 (32)
Ginn, 2005^48^	0	0	0	1	0	0	1	0	0	1	1	0	1	0	1	0	0	0	0	6 (32)
Giombini, 2006^59^	1	0	0	1	0	0	0	0	0	0	1	1	1	0	0	0	0	0	0	5 (26)
Haahr, 2005^49^	0	0	0	0	0	0	0	0	0	1	0	0	1	0	0	0	0	0	0	2 (11)
Hay, 2003^50^	0	0	1	0	0	0	0	0	0	0	1	0	1	0	0	0	0	0	0	3 (16)
Holmgren, 2012^36^	1	0	0	1	1	0	0	1	1	1	1	0	1	1	1	1	0	0	0	11 (58)
Kachingwe, 2008^51^	0	1	1	1	0	0	0	0	0	1	1	0	0	0	0	0	0	0	0	4 (21)
Littlewood, 2014^52^	1	0	0	0	1	1	1	1	0	0	1	0	1	1	1	1	1	0	1	12 (63)
Lombardi, 2008^53^	0	0	0	0	0	0	1	1	0	0	1	0	0	0	0	0	1	0	0	4 (21)
Ludewig, 2003^60^	1	0	0	1	0	0	1	1	1	1	0	0	0	0	1	1	0	0	0	8 (42)
Maenhout, 2013^61^	1	0	0	1	0	0	1	1	1	1	1	0	0	1	1	1	0	0	0	10 (53
Martins, 2012^62^	1	0	0	0	0	0	0	0	0	0	0	0	0	0	0	0	0	0	0	1 (5)
Marzetti, 2014^40^	1	0	0	0	0	0	0	1	0	0	0	0	1	0	1	1	0	0	0	5 (26)
Moosmayer, 2014^54^	0	0	0	1	0	0	0	0	0	1	1	0	1	0	1	0	0	0	0	5 (26)
Østeras, 2008^63^	1	0	1	1	0	1	0	1	0	0	0	0	0	0	1	1	1	0	0	7 (37)
Rhon, 2014^55^	1	1	1	0	0	0	0	0	1	1	1	1	1	0	1	0	0	1	0	10 (53)
Şenbursa, 2011^56^	0	0	0	1	0	0	0	0	0	1	1	0	0	0	0	0	0	0	0	3 (16)
Struyf, 2013^41^	0	0	0	0	0	0	0	0	0	1	0	0	0	0	1	0	0	0	0	2 (11)
Subasi, 2012^64^	0	0	0	1	0	0	0	0	0	1	1	0	0	0	0	0	0	0	0	3 (16)
Szczurko, 2009^65^	0	0	0	0	0	0	0	0	1	0	1	0	0	0	0	0	0	0	0	2 (11)
Walther, 2004^66^	1	0	0	1	0	0	0	0	1	1	1	0	0	0	1	1	0	0	0	7 (37)
Wang, 2006^67^	0	0	0	1	1	0	1	1	0	0	0	0	1	0	1	1	0	0	0	7 (37)
Winters, 1997^44^	0	0	0	1	0	0	0	0	0	0	1	0	0	0	0	0	0	0	0	2 (11)
Total	13 (38)	4 (12)	7 (21)	21 (62)	5 (15)	3 (9)	9 (26)	14 (41)	10 (29)	17 (50)	23 (68)	4 (12)	16 (47)	7 (21)	17 (50)	10 (29)	4 (12)	2 (6)	2 (6)	

CERT, Consensus on Exercise Reporting Template.

**Figure 2:**
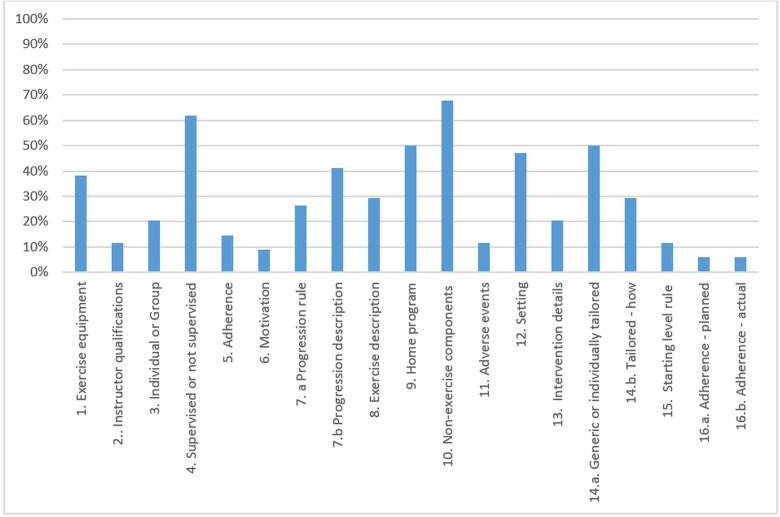
Percentage of interventions (out of 34) with complete reporting for each of the Consensus on Exercise Reporting Template checklist.

Four CERT items were clearly described by at least 50% of the trials (figure 2). These included description of any non-exercise component (23 trials), whether the intervention was supervised or not supervised (21 trials), if the intervention included a home programme (17 trials), and whether it was generic or individually tailored (17 trials). The following items were particularly poorly reported across most trials: motivational strategies were described in only three trials (9%)^34 52 63^; how adherence or fidelity was assessed/measured was reported in only two trials (6%)^34 55^; and to what extent the intervention was delivered as planned was reported in only two trials (6%).^35 52^

### Inter-rater agreement of CERT assessment

Table 3 presents percentage agreement and PABAK coefficients for each CERT item. For a majority of the items, inter-rater agreement was high according to percentage agreement (15/19 items: >80%) and it was substantial (11 items: 0.61–0.80) or excellent (5 items: 0.81–1.0) according to the PABAK. There was acceptable agreement for the descriptions of the type of exercise equipment (item 1) (74% agreement, PABAK 0.47); qualifications (item 2) (71% agreement, PABAK 0.41); and which setting the exercises were performed (item 12) (79% agreement, PABAK 0.59).

**Table 3:** Percentage agreement and inter-rater reliability for each CERT item

CERT item	% Agreement by item*	Strength of agreement	PABAK kappa coefficient† (95% CI)	Strength of the agreement‡
1. Equipment	74	Acceptable	0.47 (0.29 to 0,76)	Moderate
2. Instructor qualifications	71	Acceptable	0.41 (0.07 to 0.76)	Moderate
3. Individual or group	88	High	0.76 (0.48 to 1.0)	Substantial
4. Supervised or not supervised	88	High	0.76 (0.51 to 1.0)	Substantial
5. Adherence	91	High	0.82 (0.52 to 1.0)	Excellent
6. Motivation	97	High	0.94 (0.47 to 1.0)	Excellent
7a. Progression rule	88	High	0.76 0.46 to 1.0)	Substantial
7b. Progression description	88	High	0.76 (0.48 to 1.0)	Substantial
8. Exercise description	91	High	0.82 (0.54 to 1.0)	Excellent
9. Home programme	82	High	0.65 (0.38 to 0.92)	Substantial
10. Non-exercise components	82	High	0.65 (0.36 to 0.93)	Substantial
11. Adverse events	91	High	0.82 (0.36 to 1.0)	Excellent
12. Setting	79	Acceptable	0.59 (0.28 to 0.89)	Moderate
13. Intervention details	85	High	0.71 (0.38 to 1.0)	Substantial
14a. Generic or individually tailored	79	Acceptable	0.76 (0.3 to 0.88)	Substantial
14b. Tailored how	88	High	0.76 (0.46 to 1.0)	Substantial
15.Starting level rule	88	High	0.76 (0.34 to 1.0)	Substantial
16a. Adherence (planned)	88	High	0.76 (0.7 to 0.83)	Substantial
16b. Adherence (actual)	94	High	0.88 (0.41 to 1)	Excellent

*For percentage agreement scores, the strength of agreement is expressed by the following descriptors:<70%=poor, 70%–79%=acceptable and ≥80%=high.

†Inter-rater reliability measured using the PABAK coefficient.

‡For PABAK scores, the strength of agreement is expressed by the following descriptors: 0=poor, 0.01–0.20=slight, 0.21–0.40=fair, 0.41–0.60=moderate, 0.61–0.80=substantial and 0.81–1=excellent.

CERT, Consensus on Exercise Reporting Template; PABAK, prevalence and bias adjusted kappa.

Consensus was reached on all the dissonant items without the need for an independent arbiter. The reason for the disagreements on these items was that one of the reviewers had a stricter interpretation of the CERT Explanation and Elaboration Statement than the other reviewer (items 1, 2 and 9) and reviewer error (item 12). For item 1 (equipment), the authors had described most of the equipment used, but a detailed description was missing for one or more of the exercises. The disagreement on item 2 (qualifications) was because one of the reviewers rated descriptions such as ‘experienced physiotherapist’, ‘highly experienced physiotherapist’ and ‘musculoskeletal physiotherapist’ as clearly described, while the other reviewer had a stricter interpretation of the item and argued that the description of qualification should also include duration of experience. The seven disagreements on item 12 (setting) was caused by reviewer errors, where the setting had been clearly described in the main paper or the protocol, but one of the reviewers had missed it. For the disagreements other than those that were considered errors, the authors chose to agree on the strictest interpretation in all occasions.

## Discussion

The main result of this paper is that trials investigating exercise interventions designed for patients with rotator cuff disease poorly describe the content of their exercise interventions. Most CERT items were not described in enough detail to be able to be replicated, while only four CERT items were clearly described by at least 50% of the included trials. Overall, our results also indicate that the inter-rater reliability of the CERT is high/acceptable.

### Comparison with other studies

Our finding of incomplete descriptions of exercise interventions in our corpus of trials is in keeping with previous studies that have evaluated exercise descriptions using the CERT in trials in knee osteoarthritis and a random sample of musculoskeletal exercise trials.^15 17^ Comparable findings were also found in a systematic review that assessed the content reporting for exercise interventions for patellofemoral pain syndrome using the TIDieR checklist,^16 22^ and Toigo and Boutellier mechanobiological exercise descriptors.^68^ In contrast to our study, which included a random sample of rotator cuff disease exercise trials, O’Neil *et al* included only knee osteoarthritis exercise trials that had a Physiotherapy Evidence Database (PEDro) Scale total score of ≥6 out of a maximum of 10.^15^ The PEDro score evaluates risk of bias as well as adequacy of trial reporting. Based on the CERT scores of each included study reported in O’Neil *et al*’s supplementary material (appendix 1), we calculated that the included trials had an overall median CERT score of 11 (range 4–17). The higher median score may indicate reporting of exercise interventions is better in higher quality trials.

This is also indicated by our results where the three trials judged to be at low risk of bias were among the six trials with a CERT score of ≥10. A post hoc analysis, requested by a reviewer, also revealed a statistically significant (p=0.026), weak positive correlation (Spearman’s r=0.38) between the CERT score and the number of low bias items on the Cochrane risk of bias tool. Other possible explanations for the higher CERT score could also be that the content reporting of exercise interventions in trials for people with knee osteoarthritis are generally better than trials for people with rotator cuff disease, or that O’Neil *et al* were less strict when applying the CERT.

Overall, we determined that the inter-rater reliability of the CERT was high/acceptable by two different agreement measures, and this is keeping with the recently published study by Slade *et al*.^17^ However, there are some minor differences across studies for some specific items. We found acceptable/moderate agreement for the CERT item concerning qualifications (item 2), whereas Slade *et al*^17^ reported high/excellent inter-rater reliability for this item. This may have been due to lack of clarity that duration of experience is an important consideration, and this needs to be made explicit in the CERT. Slade *et al*^17^ reported poor/fair inter-rater reliability for item 14b (tailored—how) and item 15 (starting rule), whereas we reported high/substantial inter-rater reliability for the same items. The better reliability of these latter items in our study is likely explained by the clarification of how these items should be assessed following the study by Slade *et al.*^17^

### Strengths and limitations

A strength of our study was the use of an internationally endorsed reporting guideline for assessing the completeness of descriptions of exercise interventions in clinical trials. The CERT has previously been shown to be user-friendly and time-efficient for review purposes.^17^ In order to ensure similar interpretation of the CERT items, we pilot tested the extraction form for familiarisation purposes before assessing the included trials. We assessed a sample of rotator cuff disease trials investigating the value of exercise from a recent Cochrane Review. This ensured that the included studies were relevant and important for the shoulder research field. Since all the included studies were published prior to the CERT or TIDier, this study reflects the practice of content reporting of exercise interventions without any influence of the new intervention reporting recommendations. Finally, the inter-rater agreement may also have been overestimated due to the small sample size of 34 and the precision of the PABAK estimates, which might have been higher with more studies.

### Implications for practice

To be able to interpret, use, or replicate the research, published reports need to include a sufficiently clear, complete and accurate description of the intervention.^18^ If clinicians, patients and policy makers are to implement the latest evidence from systematic reviews and/or trials, they need to be able to access clearly described interventions that include all the necessary detail about the specific components of an exercise programme. Our results indicate that only 2 of 34 trials reported whether the intervention was delivered as planned, and only 5 trials reported how they assessed/measured adherence. Not reporting these items hinders accurate interpretation of trial findings, as the reader does not know if the patients/therapists did more/less than what was described in the intervention and/or if the participants adhered to the exercise intervention. Incomplete reporting is also challenging for meta-analysis, which may lead to inappropriate pooling of data from heterogeneous interventions and, in worst case scenarios, erroneous conclusions.

### Implications for research

Detailed description of each CERT item would enable researchers to conduct further research in order to identify which items are (or are not) significantly associated with the outcome (eg, Does the qualification of the instructor matter?). This could inform future exercise interventions.^69^ Trial authors should be obliged to provide a detailed description of their intervention either in the primary paper reporting their results or in a protocol paper. Where further details are provided elsewhere, it is important that these are available via open access and that links remain unbroken over time. The use of the CERT has the potential to advance this field for researchers, peer reviewers, policymakers and clinicians by facilitating an unambiguous description of exercise programmes, which would ultimately lead to less research waste and more timely uptake of evidence into practice.^70^

## Conclusion

The completeness of content reporting of exercise interventions in RCTs of trials for people with rotator cuff disease is poor. The CERT is a reliable tool to evaluate the completeness of reporting of exercise interventions in trials. We strongly encourage journals to mandate the use of the CERT when reporting the protocols and results of trials investigating exercise interventions.
